# The Features of Beta-Amyloid Phosphorylation in Alzheimer’s Disease

**DOI:** 10.32607/actanaturae.27456

**Published:** 2024

**Authors:** P. A. Strelnikova, A. E. Bugrova, N. V. Zakharova, K. V. Danichkina, M. I. Indeykina, M. S. Gavrish, V. G. Krut’, A. A. Babaev, A. Yu. Morozova, A. S. Kononikhin, V. A. Mitkevich, A. A. Makarov, E. N. Nikolaev

**Affiliations:** Skolkovo Institute of Science and Technology, Moscow, 121205 Russian Federation; Emanuel Institute of Biochemical Physics, Russian Academy of Science, Moscow, 119334 Russian Federation; Institute of Neuroscience, Lobachevsky State University of Nizhny Novgorod, Nizhny Novgorod, 603022 Russian Federation; Pirogov Russian National Research Medical University, Moscow, 117997 Russian Federation; V. Serbsky National Medical Research Center of Psychiatry and Narcology, Moscow, 119034 Russian Federation; Mental Health Clinic No. 1 named after N.A. Alekseev, Moscow Healthcare Department, Moscow, 117152 Russian Federation; V.L. Talrose Institute for Energy Problems of Chemical Physics, N.N. Semenov Federal Research Center for Chemical Physics, Russian Academy of Sciences, Moscow, 119334 Russian Federation; Engelhardt Institute of Molecular Biology, Russian Academy of Sciences, Moscow, 119991 Russian Federation

**Keywords:** Beta-amyloid, mass spectrometry, Alzheimer’s disease, phosphorylation

## Abstract

Accumulation of neurotoxic aggregates of beta-amyloid peptides (Aβ) is a hallmark of Alzheimer’s disease (AD) progression. Post-translational modifications (PTMs) increase Aβ aggregation and cytotoxicity, and the content of specific Aβ proteoforms is elevated in senile plaques of AD patients. The pathophysiological mechanisms of aggregate formation and the role of Aβ proteoforms need thorough study both to understand the role played by specific processes in the initiation of neuronal degradation and to find effective preventive means of therapeutic action. The present work investigates the dynamics of accumulation of phosphorylated serine-8 proteoform Aβ (pSer8-Aβ) using the 5xFAD mouse amyloid model. Aβ samples from human cerebrospinal fluid (CSF) and brain were also investigated. Western blot studies using 1E4E11 and 4G8 antibodies showed that accumulation of pSer8-Aβ in mouse brain starts as early as at the age of 3 months and reaches a maximum by the age of 14–17 months, which is generally similar to the dynamics of accumulation of the total pool of Aβ peptides. The pSer8-Aβ level in human CSF in AD patients can reach ~ 1–10% of the total amount of Aβ. Mass spectrometric analysis showed that Aβ phosphorylation by the Ser8, Tyr10, and Ser26 residues in brain tissues, as well as phosphorylation of the APP by Thr719 residue, is possible. These findings support the assumption that pSer8-Aβ proteoforms are involved in amyloidosis in AD. KEYWORDS Beta-amyloid, mass spectrometry, Alzheimer’s disease, phosphorylation.

## INTRODUCTION

Alzheimer’s disease (AD) accounts for 60–80% of all dementia cases, the risk of developing the condition being particularly high amongst the elderly [1]. The number of AD patients continues to increase significantly with every passing year and may reach 115 million by 2050 [2]. The pathogenesis of AD is closely related to the homeostasis of the beta-amyloid peptides (Aβ) in the brain [3], and amyloid deposits are recognized markers of AD. Despite the more than 30 years of research history into this condition [4, 5], the need to elucidate detailed aspects of the molecular mechanisms of amyloidosis remains relevant, since influencing amyloidosis seems to be the most obvious strategy in the search for effective therapeutic agents for AD [6, 7, 8].

The full-length forms of Aβ peptides (Aβ1-40 and Aβ1-42) have important physiological functions that may vary in different tissues of the body, where these peptides take form from different isoforms of the amyloid precursor protein (APP), with the participation of β- and γ-secretases [9, 10]. These peptides play an important role in the regulation of angio- and neuro- genesis, as well as the reduction of the permeability of the blood–brain barrier (BBB). They also promote post-traumatic brain recovery [11]. An ability to aggregate to form cross-β structures is an important feature of Aβ peptides [12]. A lack of balance between the formation of full-length Aβ forms and their timely degradation can contribute to the formation of the modified proteoforms that form neurotoxic oligomers and aggregates [3, 13]. In particular, delayed degradation of full-length Aβ-peptides leads to the accumulation of truncated proteoforms [14, 15, 16, 17] and emergence of post-translational modifications (PTMs), which stabilize Aβ aggregates and promote their further growth. It is noteworthy that most of the aforementioned modifications are located in the zinc-binding domain and refer to six polar amino acid residues in the N-terminal region 1–11 [17]; the presence of characteristic peptides truncated at the N-terminus in brain amyloid plaques is consistent with the increased modification rate in this region [15].

In general, pyro-Glu3, pyro-Glu11, isoAsp1, and isoAsp7 are the most abundant PTMs of Aβ peptides [14, 18, 19, 20, 21, 22, 23], a fact that may contribute to their increased aggregation and the stabilization of senile plaques in AD. In particular, the assessment of the dynamics of accumulation of different PTMs in the amyloid deposits in the brains of 5xFAD mice used as AD models showed that the accumulation of pyro- Glu3-Aβ in these mice starts at a very early age and reaches a plateau by 8 months of age, whereas isomerization of Asp7 at 7 months of age amounts to only 8% and reaches its maximum (~30%) by the end of the life cycle, but never reaching saturation [24].

Ser8 phosphorylation is another PTM of the N-terminal Aβ site that may play an important role in AD pathogenesis. In particular, the formation of pSer8-Aβ, which occurs with participation of the protein kinases in the extracellular space and on the surface of brain cells [25], correlates with the manifestation of AD symptoms and impedes the Aβ degradation involving insulin-degrading and angiotensin- converting enzymes [26]. The immunohistochemical analysis of neocortex samples revealed the existence of a relationship between the phosphorylation of Aβ by Ser8 and its aggregation into dispersible oligomers, protofibrils and fibrils in symptomatic AD, but not at the preclinical stage [27]. Using synthetic full-length pSer8-Aβ1-42 peptides, rapid formation of stable fibrillar aggregates and neurotoxic oligomers was observed in the absence of zinc ions [28, 29]. In addition, the possibility of formation of aggregates exhibiting increased neurotoxicity due to the interaction of pSer8-Aβ1-42 with unmodified Aβ1-42 was established [30]. The structural NMR analysis additionally revealed the higher amyloid amplification efficiency and increased thermodynamic stability of pSer8-Aβ1-40 fibrils compared to the fibrils of the unphosphorylated peptide [31]. On the other hand, both full-length pSer8-Aβ1-42 and its zinc-binding fragment pSer8-Aβ1-16 significantly reduce the *in vitro *Aβ aggregation induced by zinc ions [32, 33, 34] and injections of pSer8-Aβ1-42 reduce the formation of amyloid plaques in the mouse hippocampus [34]. Apparently, both scenarios are possible, when pSer8-Aβ triggers fibril formation or, on the contrary, supresses metal- dependent aggregation. The realization of each scenario depends on external factors such as the presence and concentration of zinc ions, Aβ partner proteins, etc. In this regard, studying the dynamics of pSer8-Aβ accumulation may help reaseachers clarify its role in the formation of amyloid deposits.

The optimization of approaches, including mass spectrometric (MS) ones, to the analysis of phosphorylated forms of amyloid remains a pressing task [17, 35, 36]. Aβ includes three potential phosphorylation sites (Ser8, Ser26, and Tyr10), as well as potential sites in the APP regions adjacent to Aβ. Evidence has been obtained of the *in vivo *presence of phosphorylated Aβ proteoforms with modified Ser8 and Ser26 [17, 24]. Further MS studies of these proteoforms may lead to the uncovering of important information on the role of phosphorylation pathways in amyloidosis, as well as to our understanding of the role played by phosphorylation in the pathogenesis of AD. In this work, we investigated the specificity of Aβ phosphorylation by Ser8 (pSer8-Aβ) using mass spectrometry and Western blotting in 5xFAD model mice, as well as in an AD patient.

## EXPERIMENTAL

**Reagents and Aβ-peptides**

All the chemicals and solvents used in this study were of HPLC grade. Synthetic Aβ peptides (Aβ_1-16_, pSer8-Aβ_1-16_, Aβ1-42 and pSer8-Aβ_1-42_) prepared by solid- phase synthesis and purified to > 99.5% purity using HPLC (BioPeptide Inc., USA) were dissolved in 10% acetonitrile to a concentration of 0.5 mg/mL, divided into 50 μL aliquots, and stored at -80°C. The synthetic peptides at known concentrations were used as standards and control samples (“spikes”). For the control samples, human blood plasma diluted with phosphate buffered saline (pH 7.4) at a 1 : 50 ratio was used as a template.

**5xFAD mice**

The 5xFAD transgenic mouse line (Jackson Laboratory, USA, stock number 006554) was used in this work [37]. Mice of this line have five severe AD-related inherited mutations in the human amyloid precursor protein (APP) (SweK670N, M671L, LonV717I and FloI716V) and the presenilin 1 (PSEN1) (M146L and L286V) genes expressed under the control of the mouse Thy1 promoter. These mice express transgenic human Aβ at significantly higher levels than native mouse Aβ, resulting in an accelerated deterioration of the cognitive brain function. The earliest behavioral impairments manifest themselves at an age of ~ 6 months and become maximally pronounced by 9 months of age [38].

Mouse brain samples were provided by the Center for Genetic Collections of Laboratory Animals (Lobachevsky Institute of Biology and Biomedicine, NNGU). The cohort consisted of six items aged 3, 8, 12, 12, 14, 17, and 23 months. The animals were kept in a colony with free access to food and water in a room with controlled temperature +20 ± 2°C and 40–60% humidity with a 12-hour light/dark cycle. The experiments on mice were performed according to the guidelines approved by the Local Ethics Committee and animal control authorities (Guidelines for Housing and Care of Animals. Environment, Housing and Management, 2016).

All the 5xFAD mice were genotyped to confirm the presence of all mutations. For this purpose, DNA was extracted from part of the tail, placed in 300 μL of lysis buffer (10 mM Tris-HCl, pH 8.0, 100 mM NaCl) with proteinase K, incubated at +55°C for 16–20 h with rotation at 650 rpm, mixed, and centrifuged at room temperature (5 min, 14 000 rpm). The upper fraction containing DNA was additionally washed once with isopropanol and twice with 80% ethanol, followed by centrifugation at +4°C (15 min, 14 000 rpm). The presence of the human* PSEN1 *and *APP *genes was verified by polymerase chain reaction (PCR) using the insert-specific primers 5′-AAT AGA GAA GAA CGG CAG GAG CA-3′ and 5′-GCC AT G AGG GCACT AAT CAT-3′ for* PSEN1 *and 5′-AGG ACT GAC CACT CG ACC AG-3′ and 5′-CGG GGGTCTAGTTCTTCTGCAT-3′ for *APP* [39]. The control primers were 5′-CT A GGC CAC AGAATTGAAAGATCT-3′ and 5′-GTAGGTGTGGA AAT T CT AGC ATC C-3′.

**Human CSF and brain samples**

A human brain tissue sample was obtained from an 82-year-old sporadic AD patient at autopsy (post-mortem interval (PMI), 15 h). Samples from the temporal lobe and hippocampus were frozen in liquid nitrogen for further MS analysis and fixed in 10% buffered formalin (BioVitrum, Russia) for histological analysis. To verify the diagnosis, 4–5 μm thick histological sections of brain tissue were made and stained with Congo red (BioVitrum, Russia). Anti-tau-protein and anti-Aβ antibodies were used for immunohistochemical (IHC) staining.

A CSF sample was obtained by diagnostic lumbar puncture with a sterile needle from a 79-year-old man diagnosed with Alzheimer’s. The volume of cerebrospinal fluid was 1 mL. The sample was centrifuged (1 500 rpm, 15 min) within 1 h of collection, and the supernatant was collected. The samples were stored at -80°C.

All the procedures related to the obtention of human biological material were performed in accordance with the recommendations of the Declaration of Helsinki and after securing the approval of the Ethics Committee (Protocol No. 1 of January 25, 2022 of the Ethics Committee of the Mental Health Clinical Hospital No. 1 named after N.A. Alekseev, Moscow City Health Department). Written informed consent was obtained from the patients.

**Extraction of Aβ-peptides from the brain tissue**

For Aβ-peptide extraction, brain samples were homogenized on ice using a Potter glass homogenizer with the addition of four volumes of lysing buffer (20 mM Tris, 2.5 mM EDTA, 137 mM NaCl, pH 7.6) containing a mixture of protease inhibitors (Roche, France). After formic acid (FA) had been added up to 70% (by volume), the samples were sonicated (10 min × 2), shaken, and centrifuged (30 000 g, 1 h, +4°C). Evaporation of FA from the supernatant was achieved using a vacuum concentrator (Eppendorf, Germany). The obtained acidic extracts were stored at -80°C. Aβ was further isolated by solid-phase extraction (SPE) using Oasis MCX cartridges (Waters, USA) according to the manufacturer’s protocol [40], with the following modifications: the acidic extracts were dissolved in a 8 M/2 M urea/thiourea mixture, and H3PO4 was added up to 2%. SPE cartridges were preconditioned with 1 mL of methanol and equilibrated with 1 mL of 4% H3PO4. The sample (2 mL) was loaded into the SPE cartridge and washed with 2 mL of 4% H3PO4 and 10% acetonitrile (ACN). The Aβ-enriched fraction was eluted with 1 mL of a 75 : 15 : 10 (vol.) ACN/H_2_O/ NH_4_OH solution, and the samples were evaporated to 100 μL using a vacuum concentrator (Eppendorf, Germany).

**Aβ-peptide isolation from human CSF**

Aβ-peptides were isolated from CSF and the control samples (“spikes”) by immunoprecipitation (IP) using the monoclonal antibody 1E4E11 highly specific to pSer8-Aβ (Merck KGaA, Germany), as well as the antibodies 6E10 and 1E8 (three orders of magnitude more sensitive to unmodified Aβ) and 4G8 (with equal specificity to both forms of Aβ). IP was performed for 3 h at +4°C with the antibodies immobilized on Dynabeads magnetic particles (Thermo Scientific) in the presence of protease inhibitors. Aβ was eluted using 70% ACN supplemented with 10 mM HCl or double-denaturing buffer for samples for the subsequent peptide electrophoresis and Western blotting.

**Peptide electrophoresis and Western blotting**

The samples obtained after SPE enriched with the Aβ fraction were mixed at a 1 : 1 ratio with 2× sample buffer for peptide electrophoresis (100 mM Tris-HCl pH 8.8, 1% SDS, 4% beta-mercaptoethanol, 24% glycerol, 0.02% Coomassie brilliant blue) and denatured for 5 min at ~ +90°C [41]. Peptide electrophoresis was performed in Mini-PROTEAN cells (Bio-Rad, USA) using a Tris-tricine buffer system (25 mM Tris, 25 mM Tricine, 0.05% SDS) in 12% polyacrylamide gel. Four mL of a 30% acrylamide/ bis-acrylamide solution (AA/BA, 29/1), 5 mL of 2.5 M Tris-HCl pH 8.8, 6 μL of TEMED, and 100 μL of 10% ammonium persulfate (PSA)) were added per 10 mL of separating gel. To prepare 5 mL of a 4% concentrating gel, 0.66 mL of AA/BA, 0.76 mL of 2.5 M Tris-HCl pH 8.8, 5 μL of TEMED, and 150 μL of PSA were added. Electrophoresis was performed for 1.5–2 h at 60 mA with sufficient cooling until the dye front started to emerge from the gel. Semi-dry transfer to a 0.2 μm pore size nitrocellulose membrane was performed for 1 h at 50 mA using a buffer containing 47.9 mM Tris, 38.6 mM glycine, 0.0385% SDS, and 20% methanol.

For Western blotting, the membrane was blocked for 30 min in a 2.5% milk solution in 0.01 M PBS and 0.1% Tween-20 supplemented with 10 mg/mL BSA and incubated with primary antibodies overnight. The 1E4E11 antibody (1 : 4,000, Merck KGaA, Germany), highly specific to this PTM, was used to detect pSer8-Aβ. Unmodified Aβ was detected using 6E10 antibodies (1 : 8 000, epitope 3–8, Biolegend, USA), as well as 4G8 antibodies (1 : 4 000, epitope 18–23, Biolegend, USA) capable of detecting both monomeric and oligomeric forms [42]. Incubation with secondary antibodies (1 : 5 000, horseradish peroxidase- conjugated IgG, Hy-test) was performed for 1 h. The results were visualized using enhanced chemiluminescence (ECL) with SuperSignal reagents (Thermo Fisher Scientific, USA). Images were acquired using the SYNGENE G:Box gel documentation system (Syngene, UK) and processed using the GeneTools software.

**Chromatography-mass spectrometry (HPLC-MS) analysis**

All the samples meant for HPLC-MS analysis were hydrolyzed by Lys-C protease (Promega, USA) to yield hydrophilic fragments of Aβ1-16. Samples after SPE (20 μl) were mixed (1 : 1) with 100 mM ammonium bicarbonate and 0.4 μg Lys-C, and then incubated at 37°C for 4 h.

Non-targeted HPLC-MS analysis of hydrolysate (Lys-C) samples was performed on a Dionex Ultimate 3000 HPLC system (Thermo Fisher Scientific) coupled to a TIMS TOF Pro high-resolution time-of-flight mass spectrometer (Bruker Daltonics, USA) using the parallel accumulation and sequential fragmentation (PASEF) data acquisition method in the DDA mode. The electrospray ionization (ESI) source settings were as follows: capillary voltage, 4500 V; end plate bias potential, 500 V; and dry gas flow rate, 3.0 L/min at 180°C. Measurements were carried out in the mass/charge (m/z) range of 100 to 1,700. The ion mobility range included values from 0.60 to 1.60 V·s/cm2 (1/k0, where k0 is the ion mobility). The total cycle time was set to 1.16 s, and the number of PASEF MS/MS scans was set to 10. For small amounts of samples, the total cycle time was set to 1.88 s.

For HPLC, the loaded sample volume was 1 μL per injection. HPLC was performed using an Ion Optics emitter column (C18, 25 cm × 75 μm, 1.6 μm; Parkville, Australia) by gradient elution. Mobile phase A contained 0.1% formic acid in water; mobile phase B contained 0.1% FA in acetonitrile. Separation was performed at a flow rate of 400 nL/min using a 40-min gradient from 4 to 90% of phase B.

Data from non-target HPLC-MS were analyzed using the PEAKS Studio 8.5 software (parameters: parent ion mass measurement error, 20 ppm; fragment mass error, 0.03 Da). Methionine oxidation was identified as a possible variable modification. The search was performed using the Swissprot database of human proteins. The FDR threshold for all the steps was set at 1% or lower.

## RESULTS AND DISCUSSION

**Dynamics of pSer8-Aβ accumulation in the brains of 5xFAD mice**

The formation of fibrillar Aβ structures can be detected in the brains of 5xFAD mice as young as two months- old [37]. The dynamics of accumulation of some Aβ proteoforms studied previously showed that, in general, the accumulation of Aβ proteoforms correlates with the formation of amyloid deposits [24]. We performed a series of experiments to evaluate the dynamics of pSer8-Aβ accumulation by Western blotting.

**Fig. 1:**
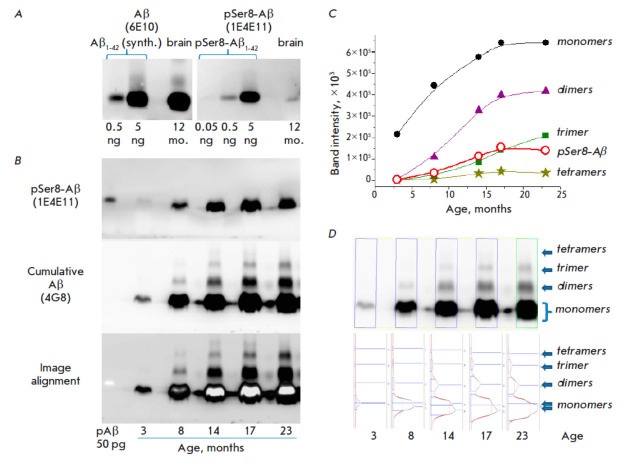
Analysis of the dynamics of pSer8-Aβ accumulation against the total pool of Aβ peptides by Western blot in the brains of 5xFAD mice. (*A*) Accumulation of unmodified and pSer8 monomeric forms of Aβ in a 12-month-old mouse brain; the amount of sample applied corresponds to 0.3 mg of brain tissue for Aβ and 1 mg of tissue for pSer8-Aβ. (*B*) Comparative analysis of the accumulation of the total pool of peptide Aβ and pSer8-Aβ forms in the brain of mice of different ages; the amount of the applied sample corresponds to 3 mg of brain tissue. (*C*) Graphs showing the dynamics of accumulation of monomeric and oligomeric forms of Aβ based on the results of image processing in the GeneTools software. (*D*) GeneTools analysis of the staining intensity distribution of bands in each lane

The proportion of phosphorylated Aβ in a middleaged mouse (12 months of age) was roughly estimated beforehand. For that purpose, we compared the peptide content in identical tissue weights from the same animal with a line of the corresponding synthetic standards Aβ1-42 and pSer8-Aβ1-42 (*Fig. 1A*). 6E10 antibodies, which are used to detect predominantly monomeric peptides (epitope 3–8), were used to identify unmodified Aβ; highly specific 1E4E11 antibodies were used to identify pSer8. The results indicate that the total amount of monomeric Ser8-unmodified Aβ proteoforms can be as high as 2 ng per mg of brain tissue by the age of 12 months, while the proportion of monomeric pSer8-Aβ can be 10–100 times smaller. It should be noted that Western blotting still does not belong to what is known as quantitative methods and allows only approximate relative quantification. In addition, both the total amount of Aβ and the amount of its modified forms can vary in different animals [24].

The dynamics of accumulation of total and modified Aβ were assessed using extracts obtained from the brain preparations of 3-, 8-, 14-, 17-, and 23-month-old mice (*Fig. 1B–D*). To minimize technical errors and, given that a preliminary experiment had confirmed that pSer8-Aβ could be present in significantly lower amounts with respect to unmodified Aβ, the same membrane was sequentially manifested first with antibodies to pSer8-Aβ (1E4E11) and only after with 4G8 antibodies, which, unlike 6E10, do not compete at all for the epitope with 1E4E11 and manifest all forms of Aβ that have no modifications in site 18–23 (including pSer8-Aβ), including their oligomers [42]. Visual evaluation of the Western blotting results (*Fig. 1B*) suggests that pSer8-Aβ peptides are already detectable at the age of 3 months and, in monomeric form, may peak by the age of 14 months. The results of additional evaluation of band intensity in the recorded images (*Fig. 1B*) demonstrate the similarity of the dynamics of pSer8-Aβ accumulation and the total pool of monomeric and dimeric forms of Aβ, which may be indirect indication of a relationship between Ser8 phosphorylation and oligomerization stimulation. Nevertheless, the result does not allow us to draw any direct conclusions about the possible presence of pSer8-Aβ in oligomeric forms: similar to 6E10 antibodies, the overlapping epitope in the N-terminus of the 1E4E11 antibodies may also poorly identify dimeric and oligomeric forms due to the reduced availability of the specific site.

Additional analysis of the distribution of the band staining intensity in each sample (*Fig. 1D*) further emphasized the presence of Aβ dimers, trimers, and tetramers in individual samples, and also revealed heterogeneity and the presence of at least two peaks in the stains corresponding to monomeric forms starting at the age of 8 months. This may indicate the presence of truncated monomers, along with fulllength peptides. Notably, when the images in *Fig. 1B* are combined, the location of the phosphorylated proteoforms can rather be confined to the upper part of the monomeric spot and, in general, the spots corresponding to pSer8-Aβ have clearer boundaries compared to the overall spot.

Hence, it can be inferred that the lower part of the total spot may contain peptides truncated at the N-terminus and lacking a Ser8 site. This may be indication that Ser8 phosphorylation may facilitate cleavage of the corresponding N-terminal fragment.

**Analysis of pSer8-Aβ in CSF**

**Fig. 2:**
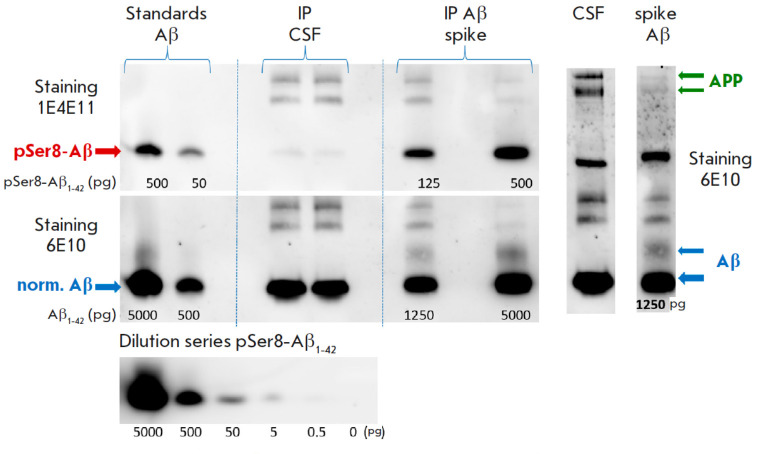
Evaluation of pSer8-Aβ content in the cerebrospinal fluid (CSF) of a patient with AD by Western blot. Immunoprecipitation (IP) was performed in the patient’s CSF, as well as the control samples of Aβ (spikes). The green arrows indicate the precursor protein (APP); the blue ones, monomer and dimer Aβ

Western blot analysis of Aβ from the CSF of a patient with AD revealed the presence of significant amounts of pSer8-Aβ (*Fig. 2*). Sequential staining of the membrane with highly specific antibodies to pSer8-Aβ and antibodies to the unmodified forms allowed us to establish that the proportion of pSer8-Aβ forms in the CSF may amount to ~ 1–10% of the unmodified fulllength forms of the peptide. This value agrees well with the results of a quantitative determination of the fraction of phosphorylated amyloid monomers obtained using the electrochemical approach [43].

In addition, in the CSF sample, antibodies against the unmodified form of Aβ identify two high-molecular- weight bands corresponding to the ARP protein (*Fig. 2*); probably its glycosylated and non-glycosylated forms.

**Mass spectrometric analysis of phosphorylated Aβ proteoforms from the brain tissue**

**Fig. 3:**
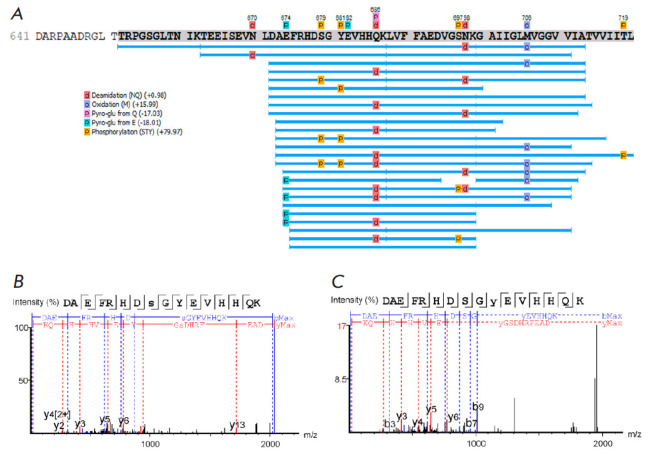
Coverage of sequences of Aβ proteoforms (marked in gray) isolated from the brains of 5xFAD mice with localization of possible post-translational modifications including phosphorylation (Ser (S), Thr(T) and Tyr (Y)) – (*A*). Fragmentation mass spectra for phosphorylated Aβ16 peptide with different localization of phospho groups (the characteristic –y –b series of peptide fragment ions are noted): spectrum for Ser8(s) – (*B*); spectrum for Tyr10(y) – (*C*)

Phosphorylation of Aβ had not previously been confirmed through mass spectrometry methods. In the present study, phosphorylation of Ser, Thr, and Tyr was examined by HPLC-MS analysis of sample fractions of Aβ from the brain tissue of 5xFAD mice (*Fig. 3*). Aβ peptides phosphorylated at the Ser8 and Tyr10 residues were identified; however, close observation of fragmentation spectra did not allow us to unambiguously determine the position of PTMs. In addition, a peptide with double phosphorylation was detected. This circumstance indicates the feasibility of the phosphorylation not only of the serine (Ser8), but also of the tyrosine (Tyr10) within amyloid. Phosphorylation of Tyr10 has been shown by neither MS nor any other method. Although it has been found that nitration and dityrosine formation can occur at this position, phosphorylation could be an intermediate reaction that is difficult to detect. In addition to Ser8 and Tyr10, some fragmentation spectra indicated the possibility of Ser26 phosphorylation. Nevertheless, the overall quality of the spectra recorded for Ser8, Tyr10, and Ser26 cannot be recognized as sufficient to draw any confident conclusions: so, this issue requires further research.

In addition, according to a HPLC-MS analysis of Aβ peptides from the human brain, phosphosite Thr (T719) was detected, whose phosphorylation was close to 100% and raised no doubt. This site is present only in elongated forms of Aβ (X- T48), which are most likely to be the products of alternative processing of APP or its degradation, unrelated to amyloidosis. Other possible phosphorylation positions in the APP — 729, 730, and 743 — had no reliable coverage in the MS analysis, making it impossible to draw any inference. The Tyr757 site had good coverage, but its phosphorylation was not detected.

In general, although the results of the MS analysis indicate the presence of certain phosphorylation sites in Aβ and adjacent APP regions, the need for further MS studies of phosphorylated Aβ proteoforms remains present. The solution to this problem may hinge to a significant extent on an optimization of the procedure for isolating phosphorylated Aβ proteoforms, since the frequently used FA solubilization readily hydrolyzes esterified phosphate groups [20, 22]. In addition, some hope is associated with the recent advances in the research into synthetic phosphorylated Aβ proteoforms by MALDI-TOF and with the use of matrix additives that minimize the loss of phosphate groups during ionization and enhance the ionization of phosphopeptides specifically [35, 36].

## CONCLUSIONS

According to the results obtained in this study, accumulation of pSer8-Aβ-peptides in the brains of 5xFAD mice follows a similar dynamics as that in the accumulation of monomeric and dimeric forms of the total pool of Aβ peptides. By the end of the life cycle, the total amount of accumulated Aβ peptides can reach ~10 ng per mg of brain tissue and the proportion of pSer8-Aβ could attain 1–10%.

It is also shown that the proportion of pSer8-Aβ-forms in human CSF may reach ~ 1–10% of unmodified full-length Aβ forms. Using high-resolution mass spectrometry, we obtained indications of the possibility of Aβ phosphorylation by the Ser8 and Ser26 residues, as well as APP phosphorylation by the Thr719 residue. Indications that Tyr10 phosphorylation might be possible have been obtained by us for the first time. Further optimization of MS techniques for the efficient analysis of phosphorylated Aβ proteoforms remains highly potent.

## Abbreviations

AD: Alzheimer’s disease
Aβ: beta-amyloid peptides
Aβ1-42: 42 amino acid long peptide
PTM: post-translational modifications
pSer8-Aβ: phosphorylated serine-8 proteoform of Aβ
CSF: cerebrospinal fluid
APP: amyloid precursor protein
SPE: solid-phase extraction
FA: formic acid
IP: immunoprecipitation
PSA: ammonium persulfate
BSA: bovine serum albumin
HPLC-MS: high-performance liquid chromatography, mass spectrometry
PASEF: parallel accumulation-serial fragmentation
